# Comparative evaluation of borneol as a context‐dependent modulator of blood-brain barrier function: barrier-restorative or permeability-enhancing agent?

**DOI:** 10.3389/fddev.2026.1934025

**Published:** 2026-10-09

**Authors:** Xiangning Yin, Sarah Ann Thomas

**Affiliations:** King’s College London, Institute of Pharmaceutical Science, London, United Kingdom

**Keywords:** blood-brain barrier, blood-tumour barrier, borneol, drug delivery, ischaemic stroke, permeability, recovery, transient

## Abstract

The blood-brain barrier (BBB) is an obstacle to effective therapy in neurological disease because it restricts drug access to the brain. Borneol, a lipophilic bicyclic monoterpene used in traditional Chinese medicine, has been associated with opposing effects on BBB function: barrier integrity restoration in ischaemic stroke models and barrier permeability enhancement in drug-delivery models. This mini-review evaluates whether these findings represent a true contradiction or a context-dependent pattern of BBB modulation. The impact of borneol treatment on the intact and damaged barrier was compared by aligning matched readouts within the physical and selective barrier phenotype categories. For example, physical barrier integrity measures explored ionic permeability, tight junction (TJ) organization and macromolecular leakage and selective barrier function measures assessed transcytosis and transport related outcomes. Molecular markers of endothelial state and extracellular matrix integrity were also compared. Across stroke models, borneol was consistently associated with improved barrier resistance, junctional structure preservation and reduced pathological leakage, with the major facilitator superfamily domain-containing protein 2a (Mfsd2a) and caveolin-1 (Cav-1)-mediated transcytosis representing the most clearly defined downstream mechanistic pathway. In delivery models, borneol was associated with transient TEER reduction, TJ protein (claudin-5) redistribution and enhanced tracer or payload passage, consistent with reversible barrier loosening. When these findings were compared through matched readouts, these differences became less contradictory and more conditionally structured. Overall, borneol is better understood as a context-sensitive modulator of BBB function rather than a uniformly barrier-opening or barrier-protective agent. However, translation remains limited by substantial heterogeneity in model, dose and timing, together with unresolved upstream mechanisms.

## 1 Introduction

The blood–brain barrier (BBB) is a specialised neurovascular interface that preserves central nervous system (CNS) homeostasis by tightly regulating the exchange of molecules between the circulation and the brain. Structurally, the BBB is formed primarily by specialised brain capillary endothelial cells joined by tight junctions (TJ) and supported by surrounding basement membranes, pericytes and astrocytic end-feet. Functionally, BBB restriction operates through three recognised barrier phenotypes. The physical barrier, formed by TJs between adjacent endothelial cells, limits paracellular diffusion of ions and small molecules. The selective barrier, comprising influx transporters such as glucose transporters (GLUT1), efflux transporters such as P-glycoprotein (P-gp), and transcytosis machinery, regulates transcellular molecular traffic and actively removes many therapeutic compounds from the CNS. The metabolic barrier, through enzymatic activity, can further modify or degrade compounds during their transit across the endothelium, although this aspect has not been systematically examined in the borneol literature and falls outside the scope of this mini review. The evidence evaluated here therefore focuses on borneol’s effects at the physical and selective barrier levels. Barrier dysfunction directly influences inflammatory cell infiltration, oedema formation and drug penetration into the brain. Modulation of BBB permeability has become an important therapeutic issue in multiple neurological disorders (Tan et al., 2023). However, the desired direction of modulation differs by disease. In ischaemic stroke, excessive barrier disruption contributes to secondary injury and poor outcome, so restoration of BBB integrity is generally beneficial. In contrast, in brain tumours such as glioma, limited drug penetration remains a major obstacle, and transient enhancement of BBB or blood–tumour barrier (BTB) permeability may improve therapeutic delivery.

Borneol is a lipophilic bicyclic monoterpene which has been widely used in traditional Chinese medicine for over two thousand years and has been used successfully in both of these apparently opposing treatment paradigms. In fact, borneol remains an essential component of classical anti-stroke formulas such as Angong Niuhuang Pill, a Qing-dynasty preparation still used for acute cerebrovascular disease (Meng et al., 2023). It is also classified as an ‘ushering drug’ within the Emperor-Minister-Assistant-Courier theoretical framework, guiding co-administered herbs toward the brain. In stroke-related models, borneol has been associated with reduced albumin leakage, lower macromolecular permeability and improved barrier integrity (Chen et al., 2019; Li et al., 2021). Importantly, in glioma-oriented delivery systems it has been shown to facilitate drug (payload) transport across in vitro BBB models (Lv et al., 2020). This creates a central controversy: the same compound appears to support barrier restoration in one pathological setting while promoting barrier passage in another (Tan et al., 2023). The key unresolved issue is therefore not simply whether borneol affects the BBB, but how its net effect becomes context-dependent and which experimental parameters should be used to define, quantify and control this effect.

This review evaluates the hypothesis that borneol is a context-dependent modulator of BBB function in ischemic stroke and brain tumour drug-delivery models, examining how experimental model, species, timing, dose and readout selection shape the interpretation of borneol as either a barrier-restorative or permeability-enhancing agent. By comparing functional, structural and supporting evidence across these contexts, this review aims to assess how far current studies move borneol from a qualitative adjuvant towards a pharmacologically controllable and clinically interpretable BBB modulator.

Articles for this minireview were identified using the PubMed database (2016–2026) and also from within these selected references. Search terms included borneol, BBB, ischaemic stroke and drug delivery. Articles in English and Chinese were considered.

## 2 Borneol in ischaemic stroke models: evidence for barrier restoration

### 2.1 BBB dysfunction in ischaemic stroke

Ischaemic stroke triggers BBB disruption through several pathways (Figure 1). Pathway 1: Adenosine Triphosphate (ATP) depletion in ischaemic lesions compromises Na^+^-K^+^ ATPase pump and Ca ^2+^-Mg ^2+^ ATPase pump activity, causing endothelial swelling and TJ destabilisation. Pathway 2: Calcium overload (Yu et al., 2019) likely activates proteases such as calpain that cleave TJ proteins (Knopp et al., 2021). Pathway 3: Reactive oxygen species (ROS) generation further damages TJ components and activates matrix metalloproteinases (MMP) that degrade the basal lamina proteins. Notably, the cellular source of MMP-9 shifts with time after ischaemia: in the early phase it is derived predominantly from infiltrating neutrophils, whereas at later stages it becomes increasingly brain-derived (Tan et al., 2023; Chen et al., 2019; Jickling et al., 2014; Wang et al., 2024). Pathway 4: Increased presence of proinflammatory cytokines (e.g., interleukin-1β (IL1β)), inflammatory cytokines (e.g., tissue necrosis factor -α (TNFα)), and endothelial cell activation (e.g., intercellular cell adhesion molecule-1 (ICAM-1) expression) alters BBB permeability. Pathway 5: Altered capillary endothelial cell signalling via the major facilitator superfamily domain-containing protein 2a (MFSD2A)-caveolin-1 (Cav-1) axis increases endocytosis and macromolecular leakage (Wang et al., 2026).

**FIGURE 1:**
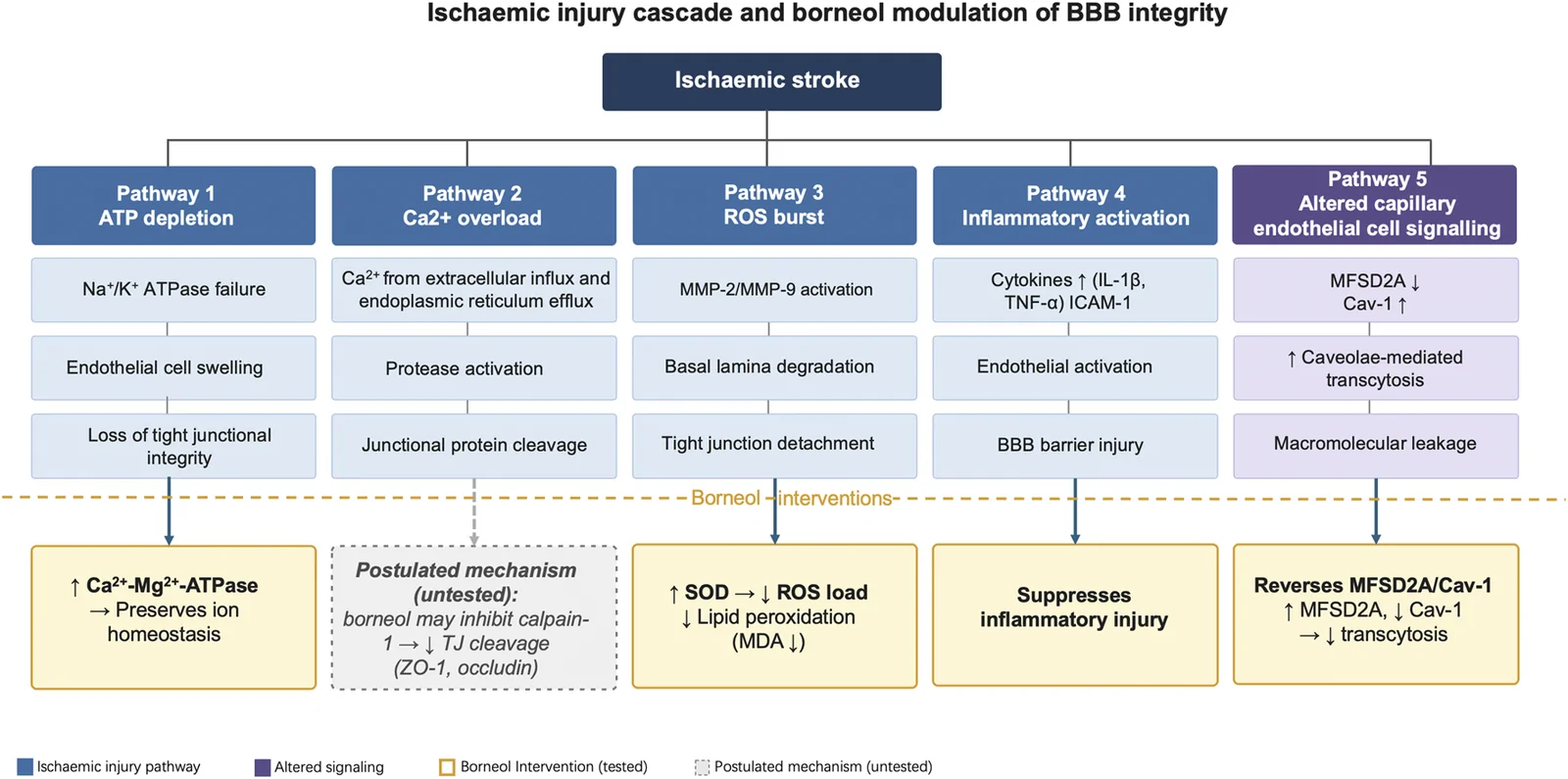
Impact of borneol on BBB dysfunction in ischaemic stroke. Ischaemic stroke disrupts BBB integrity through five interconnected pathways. Pathway 1 (ATP depletion): failure of Na^+^/K^+^-ATPase activity causes endothelial cell swelling and loss of TJ integrity; borneol partially restores ion homeostasis by upregulating Na^+^-K^+^-ATPase and Ca^2+^-Mg^2+^-ATPase activity. Pathway 2 (Ca^2+^ overload): excess intracellular calcium activates proteases that cleave junctional scaffolding proteins. Borneol reduces intracellular Ca ^2+^ overload in brain microvascular endothelial cells. A possible mechanism of action is that borneol treatment may inhibit calpain1 which is a calcium-regulated cysteine protease abnormally activated in ischaemic stroke and associated with disruption of the BBB TJ (occludin and ZO-1). Pathway 3 (ROS burst): MMP-2/MMP-9 activation degrades the basal lamina and detaches TJ; borneol upregulates superoxide dismutase (SOD), a key antioxidant enzyme that neutralises reactive oxygen species, and reduces lipid peroxidation as evidenced by decreased malondialdehyde (MDA) levels. This indicates decreased oxidative damage to the barrier. Pathway 4 (Inflammatory activation): cytokine release and ICAM-1 upregulation drive endothelial activation and barrier injury; borneol suppresses this inflammatory response. Pathway 5 (Altered capillary endothelial cell signalling): downregulation of MFSD2A and upregulation of Cav-1 promotes caveolae-mediated transcytosis and macromolecular leakage; borneol reverses this by restoring MFSD2A and suppressing Cav-1 expression. Yellow boxes indicate borneol intervention points; grey dashed box indicates absence of published endothelial evidence. Schematic of ischaemic stroke-induced BBB injury showing five pathways of damage and the proposed protective actions of borneol, including improved ion homeostasis, reduced oxidative stress, suppression of inflammatory injury, and reversal of MFSD2A/Cav-1-associated transcytosis. One pathway (Ca^2+^ overload / calpain-1 inhibition) is shown as a postulated, untested mechanism, visually distinguished from the four evidence- based intervention pathways by a dotted border and a dashed arrow.

### 2.2 Impact of borneol on BBB dysfunction in ischaemic stroke

Chen et al. (2019) reviewed fifteen preclinical studies and determined that borneol has been shown to intervene at multiple points in the ischaemic injury cascade, including restoration of ATPase activity, reducing calcium load, antioxidant enhancement and suppression of inflammatory mediators (Figure 1) (Chen et al., 2019; Yu et al., 2019; Li et al., 2021). More recently, borneol has been associated with a reduction in endocytosis as determined by vesicle number (Wang et al., 2026). These effects all reflect modulation of the injured barrier rather than thrombolytic or recanalising action on the occluded vessel. A postulated mechanism for the effect of borneol on BBB integrity is via calpain, which is a calcium-regulated cysteine protease abnormally activated in ischaemic stroke (Knopp et al., 2021) and associated with TJ (occludin and zonulae occludens (ZO-1)) disruption.

At the barrier level, functional evidence from Wang and colleagues further supports borneol’s restorative role (Wang et al., 2026). This translated to (i) reduced albumin extravasation *in vivo* assessed using the middle cerebral artery occlusion (MCAO) rat model of ischaemic stroke and (ii) reduced horseradish peroxidase transfer *in vitro* (assessed using an oxygen-glucose deprivation (OGD) immortalised human cerebral microvascular endothelial (hCMEC/D3)-pericyte co-culture model of ischaemic barrier injury). As both albumin (∼69 kDa) and horseradish peroxidase (44 kDa) are large proteins, these readouts index macromolecular permeability without, in themselves, distinguishing the paracellular from the transcellular route. Further *in vitro* studies focused on the physical barrier recovery and revealed that all tested doses of the borneol-containing preparation (6:4 L-borneol/L-canogir combination; 50–200 μg/mL) significantly restored trans endothelial electrical resistance (TEER), which is a measure of paracellular ionic permeability. Importantly though horseradish peroxidase BBB transfer studies decreased significantly only at the highest dose (Wang et al., 2026). This dissociation suggests that ionic barrier tightness recovers more readily than macromolecular exclusion under borneol treatment and indicates the involvement of both the paracellular and transcellular pathways in BBB integrity loss in ischaemic stroke models. At the level of the selective barrier, the most mechanistically defined pathway links borneol to the suppression of caveolae-mediated transcytosis via the MFSD2A and Cav-1 signalling axis. MFSD2A is a docosahexaenoic acid transporter that maintains the distinctive lipid composition of brain endothelial membranes, thereby suppressing caveolae formation. Cav-1 is the structural coat protein required for caveolar vesicle assembly (Tan et al., 2023). Under ischaemic conditions, MFSD2A is downregulated and Cav-1 upregulated, leading to increased vesicular transcytosis and macromolecular leakage. In MCAO rat models of ischaemic stroke, borneol treatment reversed both Cav1 and MFSD2A expression changes and markedly reduced endothelial vesicle numbers on transmission electron microscopy (TEM), indicating suppression of this transcellular leakage route (Wang et al., 2026).

Complementary evidence from a permanent MCAO rat model demonstrated that borneol significantly reduced serum MMP-9, a protease responsible for basal lamina degradation (Ma et al., 2021), pointing to parallel protection of the extracellular matrix alongside the transcytosis pathway. Collectively, these convergent findings—spanning direct functional permeability, vesicular transport, TJ ultrastructure and matrix integrity—support a restorative role for borneol in the ischaemic BBB with the MFSD2A/Cav-1 axis providing the likely mechanistic transcellular pathway, though this pathway was initially identified through network pharmacology prediction (Wang et al., 2026) and still awaits direct molecular-level confirmation.

## 3 Borneol in drug-delivery models: evidence for permeability enhancement

In contrast to the ischaemic setting, the drug-delivery context presents the opposite therapeutic objective: the intact BBB restricts drug access to the brain, and transient permeability enhancement is therefore desirable. Preclinical pharmacokinetic studies consistently show that borneol co-administration increases brain/CSF exposure to co-delivered agents, as reflected by increased maximum brain concentrations (ranging from 26% to 155%) and area under the curve (AUC) values (ranging from 38% to 197%) for 12 compounds and different routes of administration including intragastric, intranasal, intraperitoneal and intravenous routes (Zhang et al., 2017).

Importantly, this effect has been described as dose-dependent, transient and reversible within 4–24 h (h), suggesting that borneol opens a temporary window of enhanced BBB passage rather than causing sustained barrier damage (Yin et., al 2017; Zheng et al., 2018). In rats given intragastric synthetic borneol, TEM showed widening and discontinuity of brain endothelial tight junctions after 4 h, most marked at 8 h, with junctional structure returning to the appearance of controls by 48 h (Ge et al., 2008). Junctional changes induced in MDCK monolayers by borneol-containing rabbit serum resolved within 24 h of removing the agent, indicating that the widening depends on continued exposure rather than on junctional structure injury (Chen and Wang, 2004). These findings establish borneol as a functional permeability-enhancing adjuvant and provide the pharmacokinetic and ultrastructural basis for examining its cellular and molecular mechanism in more detail.

At the level of the physical barrier, the clearest insight into the underlying mechanism comes from tight-junction dynamics. When the mouse brain endothelial cell line, bEnd.3, was treated with borneol (10 μg/mL) for 6 h, TEER decreased rapidly and significantly, most markedly at 1 h indicating acute loosening of paracellular tightness (Wu et al., 2024). By 4 h, TEER started to increase, suggesting reversibility. Crucially, total claudin-5 (CLDN-5) protein levels remained unchanged, but cytoplasmic CLDN-5 increased significantly, and immunofluorescence confirmed redistribution of CLDN-5 from cell–cell junctions into the cytoplasm. This dissociation between stable total expression and altered subcellular localisation indicates that borneol did not reduce total CLDN-5 under the reported experimental conditions. but displaces a key structural component from its functional position—a mechanism of tight junctional weakening through reorganisation rather than destruction (Wu et al., 2024).

In an *in vitro* BTB model study, borneol reduced barrier integrity, as reflected by lower TEER (Lin et al., 2022). Interestingly further assessment revealed that borneol did not inhibit P-gp activity when used alone. A study also found no significant changes in P-gp, occludin, ZO-1 or nitric oxide synthase (NOS) expression in healthy rat brain tissue after intragastric borneol treatment, although they reported increased Evans blue labelled albumin entry (0.99 ± 0.16 to 1.47 ± 0.21 μg/mL) and ICAM-1 mean density (Wu et al., 2018). ICAM-1 promotes the adherence and migration of leukocytes and increases the release of free radicals and cell toxins, which can aggravate endothelial cell damage and promote BBB opening (Wu et al., 2018). Wu et al. (2018) considered that the opening of the BBB by borneol is related to the increased expression of ICAM-1. Another study using a rat BTB permeability model revealed that borneol loosened the barrier, enhancing delivery of a chemotherapy drug (cisplatin) and this was related to reversible ZO-1 downregulation (Duan et al., 2016). Further studies demonstrated that *L*-borneol transiently opens the mouse BBB across a range of doses (100–900 mg/kg), enhances cisplatin brain penetration and improves the survival time of glioma-bearing mice (Yin et al., 2017).

Importantly, another study also demonstrated that barrier loosening translated into therapeutically meaningful drug delivery. Lv et al. (2020) measured the transport of paclitaxel-loaded nanoparticles across a brain capillary endothelial cell (BCEC) *in vitro* BBB model (Lv et al., 2020). Borneol co-treatment increased the 4 h transport ratio from 13.11% to 19.33% for PEG-PSNPs, and from 18.38% to 23.85% for CGKRK‐modified nanoparticles, in which CGKRK is a tumour-targeting peptide ligand. CGKRK-PSNPs combined with borneol having the highest BBB penetration ability (One-way ANOVA) (Lv et al., 2020). *In vivo* fluorescence imaging in U87MG glioma-bearing mice corroborated this, showing greater brain and tumour-site accumulation. This enhanced delivery was accompanied by a marked survival benefit, with mean survival time increasing from 17.4 days (saline) to 38.9 days (CGKRK-PSNP combined with borneol) (Lv et al., 2020).

Collectively, the delivery-context evidence supports borneol as a permeability-enhancing adjuvant, most likely via reversible TJ reorganisation via CLDN-5 displacement, though the upstream molecular trigger for this redistribution remains unidentified.

## 4 Discussion

### 4.1 Comparing readouts across contexts: restoration, opening and the problem of equivalence

When comparable BBB readouts are examined side by side, borneol shows a consistent bidirectional pattern across pathological contexts. Rather than producing irreconcilable results, it consistently shifts matched functional and structural outcomes in opposite directions according to barrier state, supporting its interpretation as a context-sensitive modulator of BBB function (S1 Table).

The most directly comparable functional readout is TEER. In the stroke context, borneol significantly increased TEER in OGD-injured hCMEC/D3 monolayers, indicating restoration of paracellular tightness. In the delivery context, by contrast, borneol caused a rapid decrease in TEER in intact bEnd.3 monolayers, with the lowest level observed at 1 h, indicating barrier loosening. Although these experiments were not performed in the same endothelial model, the use of the same functional readout makes the directional contrast especially informative.

A similar pattern is seen at the level of tight-junction organisation. In ischaemic models, transmission electron microscopy (TEM) showed preservation of tight-junction ultrastructure after borneol treatment, consistent with structural restoration of the injured barrier. In delivery models, however, total CLDN-5 remained stable but redistributed from cell-cell junctions into the cytoplasm, indicating junctional weakening without overt protein loss. Taken together, these findings show that borneol modifies junction-related barrier structure, but in opposite directions—toward preservation in ischaemia and toward loosening in intact restrictive endothelium.

This bidirectional pattern is further supported by macromolecular and transport-related outcomes (S1 Table). In MCAO models, albumin leakage decreased after borneol treatment, indicating reduced barrier disruption, whereas Evans blue albumin entry increased in healthy borneol-treated rats, indicating enhanced passage across an intact barrier. Likewise, endothelial vesicle numbers fell in stroke models while nanoparticle transport ratios increased in delivery models. Although these measures are not identical, they reinforce the same directional contrast already seen in the matched functional and structural readouts.

Taken together, these comparisons show that borneol’s bidirectionality is not arbitrary but conditionally structured by pathological context. The evidence becomes less contradictory, not more, when similar readouts are aligned: borneol repeatedly shifts BBB behaviour toward restoration in injured barriers and toward increased passage in restrictive barriers.

### 4.2 Mechanistic interpretation: borneol as a context-dependent modulator of BBB function

Borneol is of interest not only because it can access the CNS more readily than many therapeutic compounds, but because the evidence reviewed here suggests that it modulates BBB function. The central question is not simply whether borneol affects the barrier, but whether its apparently opposite effects depend on disease state and is a response to specific signalling variables or reflects inconsistency.

The more precisely these studies are compared by matched readouts, the less contradictory the two sides appear. In stroke-related models, borneol is consistently associated with reduced leakage, recovery of TEER, preservation of TJ ultrastructure and reduced vesicle-associated transport, all of which move the injured barrier back toward a less permeable state. In delivery models, by contrast, borneol is associated with TEER reduction, CLDN-5 redistribution and increased tracer or payload passage, shifting an intact barrier toward greater permeability. This suggests that borneol does not impose one fixed molecular response on the BBB. Instead, its effects are shaped by the pre-existing endothelial environment, with injured barriers tending toward restoration and intact barriers toward transient loosening.

Whether this trajectory is governed by inflammatory state, endothelial metabolic stress, baseline barrier integrity, or a combination of these factors is not yet determined. What the available data do suggest is that borneol’s bidirectionality is not arbitrary but signalling-dependent, and that defining the upstream regulatory basis of the response will be a key step toward transforming borneol from an empirically observed modulator into a pharmacologically predictable one.

### 4.3 Limitations and implications for controllability

While the context-dependency framework provides an explanation for borneol’s bidirectional BBB effects, several limitations constrain its translational value. The most important is not that the proposed mechanisms are incomplete, but that the upstream regulatory basis of borneol’s context-sensitive action remains unresolved. In both stroke and delivery settings, the leading mechanistic explanations are inferred from downstream readouts—such as MFSD2A/Cav-1-associated restoration in ischaemia or CLDN-5 redistribution during barrier opening—rather than from direct evidence showing how borneol engages the molecular processes that determine the response direction. As a result, the current framework is mechanistically plausible and consistent, but still inferential.

Interpretation is further complicated by study heterogeneity. Stroke and delivery studies differ in experimental models, borneol dose, formulation, treatment duration and readout, and no single study has examined borneol under both injured and intact barrier conditions within one directly comparable experimental design. This makes it difficult to isolate context-dependency as a biological rule. The evidence suggests that barrier baseline matters, but how much of the observed bidirectionality reflects a stable property of borneol or is method dependent is unknown.

At the same time, this apparent flexibility may itself be the source of borneol’s pharmacological interest. A BBB-active compound that drives the barrier in one direction may be easier to classify, but also more limited across diverse pathological settings. The challenge is not simply to understand this flexibility, but to determine whether it can be governed safely. There are significant risks associated with transient BBB opening, including neuroinflammation, leucocyte trafficking, oedema and potential exposure of healthy brain regions. One possible route may lie in combination strategies, in which borneol provides adaptive barrier modulation while other agents direct downstream effects more predictably. In fact, the future of borneol may depend less on turning it into a conventional single-direction drug than on learning how to preserve its responsiveness while achieving sufficient control for safe therapeutic use (Lin et al., 2022).

### 4.4 Conclusion

The available evidence supports the hypothesis that borneol exerts bidirectional effects on BBB function, and that these effects are not random or contradictory, but context dependent. In ischaemic stroke models, borneol is associated with reduced pathological leakage and preserved barrier integrity; in drug-delivery models, by contrast, it produces transient barrier loosening through junctional reorganisation. Comparing matched readouts, these findings support a consistent pattern of context-dependent modulation that remains mechanistically plausible yet incompletely resolved, with the upstream determinants of directionality still to be defined.

From a translational perspective, this context-sensitive flexibility is both the promise and the problem of borneol. What makes it difficult to standardise may also be what makes it pharmacologically interesting. A compound capable of shifting BBB behaviour in a therapeutically relevant direction across different pathological settings may offer advantages over rigidly unidirectional agents, but only if that responsiveness can be brought under sufficient clinical control. Future progress will depend not only on describing borneol’s effects more precisely, but on identifying the conditions under which its apparent adaptability can become a predictable and governable therapeutic property.
