# Supplementary material for: Comparative evaluation of borneol as a context‐dependent modulator of blood-brain barrier function: barrier-restorative or permeability-enhancing agent?
Source: Front Drug Deliv. 2026 Oct 9;6:1934025. doi: 10.3389/fddev.2026.1934025 (PMC13691729; doi:10.3389/fddev.2026.1934025)
Supplement: Supplementary file 1 [file Table1.docx]

**S1 Table***:* Comparison of representative BBB readouts in ischaemic stroke and drug-delivery models of borneol treatment. Readouts are grouped by functional category rather than by individual study to permit comparison across contexts. Directly matched measures, such as TEER, are distinguished from broader interpretive comparisons, such as macromolecular leakage or supporting molecular markers. Functional and structural evidence is prioritised, while molecular markers are included as supportive rather than equivalent readouts. Overall, the table summarises a consistent pattern: borneol shifts BBB-related outcomes in opposite directions depending on barrier state, promoting restoration in ischaemic injury and increased passage in delivery settings. A limitation of this comparison is that no single study directly compares identical borneol preparations under injured and intact BBB conditions using the same species/cell line, dose, exposure time, and readouts. Differences could also arise from species, immortalization, co-culture composition, baseline TEER, exposure duration, borneol preparation, or assay conditions rather than barrier state alone. *Crossed out cells indicate that the readout has not been reported in that context.* The ‘net BBB change’ column only reports confirmed visible differences or statistically significant results. 'Tracer' refers to fluorescent or colorimetric marker molecules used to assess BBB permeability (Evans Blue or albumin); 'payload' refers to therapeutic cargo (drugs or nanoparticles) intended for CNS delivery. The L- prefix distinguishes from D-borneol throughout. L-borneolum denotes the natural pharmacopoeial form of borneol derived from Blumea balsamifera; L-Z denotes a fixed-ratio combination of its principal components, L-borneol and L-camphor (6:4), as used by Wang et al., (9). Only monotherapy results are reported unless stated. Purity is reported where available.

| *Area of Investigation* | *Barrier State* | *Borneol Form, Timing , Dosage, Route of administration* | *Stroke Context* | *Drug Delivery Context* | *Readout* | *Net BBB Change* | *Strengths & Limits of comparison* |
| --- | --- | --- | --- | --- | --- | --- | --- |
| Macromolecular Permeability | **Stroke**:  BBB *in vivo*  Injured BBB.  MCAO vs healthy BBB (sham) | **Stroke**:  L-borneolum or L-Z (6:4),  200 mg/kg; Oral.  Once daily × 6 days. | Sprague Dawley rat. Albumin leakage high in MCAO vs sham, reduced after L-borneolum/L-Z treatment.  (Wang et al., 2026) |  | Albumin leakage (immunofluorescence) | **Stroke：**  ↓ albumin leakage | **Strengths:**  -Healthy BBB baseline.  -Same species and strain.  -Matched macromolecular-leakage readout.  -Directions of change in agreement with other studies. -The single-study Evans Blue finding is further corroborated on a larger scale by a meta-analysis of 8 different studies (pooled Evan’s Blue animal studies standard mean differences 5.85, p<0.00001)(Zheng et al., 2018).  **Limits**  -Tracer and quantification method differ.  -Different formulations. |
|  | **Delivery:**  BBB, *in vivo.*  Intact,  healthy brain.  Treated (Borneol) vs vehicle control (corn oil) | **Delivery:** Pure L-borneol, 10% w/v in corn oil, 2 mL/kg intragastric (dose-response arm: 2/1.25/0.5 mL/kg). Single dose;  Evans Blue given 30 min post-borneol, brain sampled 30 min later. |  | Sprague Dawley rat (male): Single oral dose, 10% borneol in corn oil, 2mL/2kg vs corn oil control.  Brain distribution studies. Evans Blue brain content higher after borneol vs control (0.99±0.16 → 1.47±0.21 µg/mL)(P<0.05).  (T. Wu et al., 2018). | Evans Blue tracer (albumin-bound assay) | **Delivery：**  ↑ albumin-bound (Evans Blue) tracer entry |  |
| In Vitro Physical Integrity | **Stroke**:  BBB, i*n vitro*  Injured,  OGD-like injury BBB model vs intact BBB model | **Stroke**: L-Z (6:4),  50/100//200 µg/mL;  L-Z incubation for 24h before the injury.  The injury itself was Na_2_S_2_O_4_, 5mmol/L, for 2h‘ | Human hCMEC/D3–pericyte co-culture BBB model. Na_2_S_2_O_4_/OGD-like injury reduces TEER; L-Z treatment partially restores TEER significantly at all doses vs model (p=0.0191, 0.0089, 0.0003 for 50/100/ µg/mL).  (Wang et al., 2026). |  | TEER measurement | **Stroke**:  ↑ TEER (tighter) | **Strengths:**  -Compared studies utilise adult brain capillary endothelium cell lines.  -Matched functional readout shows opposite shifts.  -Directions of change in agreement with other studies.  **Limits**  -Different species, co-culture vs monolayer, and borneol form/dose differ. |
|  | **Delivery:**  BBB, *in vitro* Intact, BBB model.  Borneol-treated vs untreated (control) bEnd.3 monolayer. | **Delivery:** Pure L-borneol, 10 µg/mL, 0.5/1/4/6 h. |  | Mouse bEnd.3 monolayer. TEER decreases rapidly, lowest at 1 h (<150 Ω·cm²), partial recovery by 4 h; P<0.001 at 1 h. (X. Wu et al., 2024) | TEER measurement | **Delivery**:  ↓ TEER (looser)  transient |  |
| Tight Junction | **Stroke:**  BBB, *in vivo.*  Injured  MCAO.  MCAO  (untreated) vs L-borneolum/L-Z treated, with sham as healthy baseline | **Stroke:** L-borneolum (200 mg/kg) or L-Z (200 mg/kg); oral, 200mg/kg, once daily × 6 days. | Sprague Dawley rat. MCAO damages TJ ultrastructure; treatment reduces junctional disruption. (Wang et al., 2026) |  | TEM: TJ ultrastructure | **Stroke:**  TJ ultrastructure preserved.  No change in integrity, | **Strengths**  -Tight-junction organisation shifts in opposite directions.  -Mechanistic interpretation in agreement with other studies.  -Two mechanisticslly distinct delivery-context findings: CLDN-5 redistribution and ZO-1/F-actin down-regulation.  -Both alterations would reduce barrier integrity.  -Together results strengthen the case for multi pathway TJ modulation.  **Limits**  -Qualitative (TEM) vs molecular (Western/IF) readouts are not directly equivalent.  -The CLDN-5 redistribution finding is based on a small sample (n=3), which limits the statistical robustness of this result and has not yet been independently replicated.  -The ZO-1/F-actin finding comes from a BTB rather than intact BBB model, so the two should be read as convergent rather than a direct replication of the same barrier state.  -Reversible BBB opening and TJ involvement has been observed by other groups (Chen and Wang, 2004; Ge et al., 2008; Yin et al., 2017; Wu et al., 2024), but specific involvement of ZO-1 reversibility has not yet been verified. |
|  | **Delivery**:  BBB model *in vitro*  Intact, BBB model.  Borneol-treated vs untreated (control) bEnd.3 monolayer. | **Delivery**: Pure L-borneol, 10 µg/mL, measured at 1h post-treatment. |  | Mouse bEnd.3. Total CLDN-5 unchanged, but cytoplasmic CLDN-5 increases and redistributes into the cytoplasm; significant, n=3. (X. Wu et al., 2024) | CLDN-5 localisation (Western blot/ immunofluorescence) | **Delivery:**  CLDN-5 delocalisation linked to loss of TJ integrity and increase permeability |  |
|  | **Delivery:**  BTB *in vivo*  BTB (Tumour-bearing), C6 glioma model.  Treated vs vehicle control | **Delivery:** Natural D-borneol (Purity>95%), 35 or 140 mg/kg, intragastric, single dose, vs 0.5% sodium carboxymethyl cellulose (CMC-Na) vehicle control, sampled at 9 time points (2min-8h) |  | Sprague Dawley rat, C6 glioma-bearing. ZO-1 and F-actin mRNA/protein expression significantly decreased in tumour tissue; Lowest at 30-45min); occludin and claudin-5 unchanged.  (Duan et al.,2016). | ZO-1/ F-actin expression (qPCR, ELISA, IHC) | **Delivery (BTB):**  ↓ ZO-1/ F-actin (reversible)  Linked to increase in permeability |  |
| Transport across BBB | **Stroke**:  BBB, *in vivo*.  Injured  MCAO.  Treated vs untreated. | **Stroke**: L-borneolum (200 mg/kg) or L-Z (200 mg/kg) | Sprague Dawley rat. MCAO increases endothelial vesicle number (transcytosis proxy); treatment reduces vesicle number. (Wang et al., 2026) |  | TEM vesicle number (proxy) | **Stroke:**  ↓ transcellular transport. | **Strengths**  -Barrier passage shifts in opposite directions in agreement with other studies.  -The survival benefit is the most clinically meaningful endpoint in this table.  **Limits**  -Different methods and types of analyses**.**  -Delivery-context borneol tested only as adjuvant, never alone — benefit cannot be isolated from nanoparticle/paclitaxel effect. -Reported survival benefit reflects the best- vs worst-performing arms of the 6 group design.  *-In vitro* BBB study dose higher than other i*n vitro* BBB studies reported in Table.  -Species of cell line not reported. |
|  | **Delivery**:  (i) BBB, *in vitro*  Intact BCEC Transwell (Permeability model ).  Treated vs untreated (with vs without borneol; nanoparticle carrier present in both arms.)  (ii) BTB *in vivo*  BTB-relevant *in vivo* tumour context.  CGKRK-PSNP+borneol vs saline. | **Delivery:** Pure L-borneol (purity >97%) was the co-administered adjuvant, NOT alone. (i) *In vitro*: 10 mg/mL (ii) *In vivo*: 25 mg/kg oral, 0.5 h before IV nanoparticles. 4 h transport assay. 6 group survival design; saline, Taxol, PEG-PSNPs with and without borneol, CGKRK-PSNPs with and without borneol. |  | (i) BCEC monolayer;  Borneol increases (P<0.05) nanoparticle transport ratio across BCEC Transwell BBB model. (Lv et al., 2020).  (ii)U87MG glioma-bearing nude mice.  Increase in mean survival time.  (17.4→38.9 days, log-rank P<0.001)  (Lv et al., 2020). | (i)Transwell nanoparticle transport ratio (%)  (ii) Mean survival Time | **Delivery:**  (i)↑payload transport;  (ii) survival prolonged |  |
| Molecular Markers (Supporting Evidence) | **Stroke:**  BBB, in vivo.  Injured  permanent MCAO,  Treated vs untreated. | **Stroke**: l-borneol, 0.05/0.1/0.2 g/kg in 5% Tween-80; Intragastric administration, 10 mL/kg, for 3 days. | Sprague Dawley rat. Serum MMP-9 elevated after ischaemic injury, reduced by l-borneol treatment. (Ma et al., 2021) |  | ELISA (serum MMP-9) | **Stroke**:  ↓ MMP-9. | **Strengths**  -Indirect supporting evidence.  -Reduced MMP-9 supports barrier stabilisation  -ICAM1 linked to BBB opening.  **Limits**  -Different markers, methods and types of analyses**.**  -MMP-9: Systemic marker, not BBB-specific.  -ICAM-1 increase is association only, not demonstrated causal mechanism. |
|  | **Delivery:**  BBB, *in vivo*.  Intact  healthy brain,  Treated vs untreated. | **Delivery:** Pure L-borneol, 10% corn oil, 2/1.25/0.5 mL/kg intragastric, 30 min before euthanasia. |  | Sprague Dawley rat. ICAM-1 mean density significantly higher in high/medium/low-dose borneol groups vs control (P<0.05, most pronounced in high-dose group); positive area and total density significantly higher only in the high-dose group vs control. (T.Wu et al., 2018) | Immunohistochemistry (ICAM-1) | **Delivery:**  ↑ ICAM-1 |  |
| Ca²⁺ Overload (Supporting Evidence) | **Stroke**:  BBB in vitro  Injured  OGD injury,  *in vitro* (endothelial).  Borneol monotherapy arm) vs model (OGD-injured, untreated), within a 5-group design. | **Stroke:**  Borneol 0.5 µM | **Stroke:** Borneol 0.5 µM —rat brain microvascular endothelial cells (BMECs), OGD-injured. Ca²⁺ overload significantly decreased (P<0.05), from the borneol-monotherapy arm of a 5-group design (control/model/TMPP/Borneol/TMPP+Borneol) (Yu et al., 2019).  Ca²⁺–Mg²⁺-ATPase activity in neuronal tissue (Li et al., 2021). |  | Laser confocal Ca²⁺ imaging; Ca²⁺–Mg²⁺-ATPase activity | **Stroke;**  ↓ Ca²⁺ overload | **Strengths**  -Indirect supporting evidence for mechanism to reduce BBB opening.  **Limits**  -Yu et al. (2019)'s primary aim was comparing tetramethylpyrazine phosphate (TMPP), borneol, and their combination; the borneol-alone (monotherapy) arm within this 5-group design is the specific data point cited here (0.5 µM Borneol significantly attenuated [Ca²⁺]i vs. model, P<0.05), consistent with this table's monotherapy-only convention.  -Li et al., 2021, systematic review) is secondary, not primary, evidence. |
